# Varied functions of immune checkpoints during cancer metastasis

**DOI:** 10.1007/s00262-020-02717-2

**Published:** 2020-09-09

**Authors:** Ali Safarzadeh, Mohsen Alizadeh, Fatemeh Beyranvand, Reza Falavand Jozaaee, Khalil Hajiasgharzadeh, Amir Baghbanzadeh, Afshin Derakhshani, Antonella Argentiero, Behzad Baradaran, Nicola Silvestris

**Affiliations:** 1 Immunology Research Center, Tabriz University of Medical Sciences, Daneshghah Ave, 5166614766 Tabriz, Iran; 2 Student Research Committee, Lorestan University of Medical Sciences, Khorramabad, Iran; 3 IRCCS IstitutoTumori “Giovanni Paolo II” of Bari, Bari, Italy; 4 Department of Biomedical Sciences and Human Oncology, University of Bari “Aldo Moro”, 70124 Bari, Italy

**Keywords:** Immune checkpoints, Metastasis, Tumor, Immune system

## Abstract

Immune checkpoints comprise diverse receptors and ligands including costimulatory and inhibitory molecules, which play monumental roles in regulating the immune system. Immune checkpoints retain key potentials in maintaining the immune system homeostasis and hindering the malignancy development and autoimmunity. The expression of inhibitory immune checkpoints delineates an increase in a plethora of metastatic tumors and the inhibition of these immune checkpoints can be followed by promising results. On the other hand, the stimulation of costimulatory immune checkpoints can restrain the metastasis originating from diverse tumors. From the review above, key findings emerged regarding potential functions of inhibitory and costimulatory immune checkpoints targeting the metastatic cascade and point towards novel potential Achilles’ heels of cancer that might be exploited therapeutically in the future.

## Background

Cancer is one of the most meaningful threatening diseases for human health and despite the endless efforts that took place during the lasts decades, the number of cancer victims reaches millions [1]. One of the most important causes of cancer mortality is metastasis, which is defined as the movement of cancerous cells from their primary sites toward other organs [2]. All the tumor cells would not metastasize because the intrinsic properties of tumor cells and the tumor microenvironment factors should move toward promoting the tumor metastasis [3]. The tumor microenvironment is comprised of a myriad of interactions between immune and tumor cells, which eventually, promote the immune system’s responses against the tumor cells through the regulation of inhibitory and costimulatory responses [4]. Immune checkpoints play substantial roles in self-tolerance as immunity regulators, which hinder the immune system’s attack against healthy cells and lower the risk of autoimmunity developing [5, 6]. T cell responses that play significant roles in the detection and eliminating of tumor cells are initiated through the detection of antigens by T cell receptors (TCRs) and are regulated through the making balance between inhibitory and costimulatory signals or immune checkpoints [7, 8]. Immune checkpoint receptors such as programmed cell death protein 1 (PD1) inhibit the activities of effector T cells and tumor cells by expressing these molecules can impede anti-tumor responses of the immune system [5].

Nowadays, immune checkpoint therapy is placed as a cancer therapy besides radiotherapy, chemotherapy, and surgery. Immune checkpoint inhibitors (ICIs) targeting the regulatory pathways of T cells to augment anti-tumor responses have led to remarkable clinical advances and developed a novel weapon for the elimination of tumors [9]. After the notable achievements for cancer therapy by the use of blocking the CTLA-4 and PD-1, which are the first detected immune checkpoints, a new surge of explorations for cancer therapy based on the blocking of immune checkpoint ligands and receptors, was emerged [10]. To date, the U.S. Food and Drug Administration (FDA) approves several drugs designed to target immune checkpoint ligands for cancer treatment. Despite, an improvement of the global conventional toxicity over the chemotherapeutic agents, ICIs point out novel immune-mediated adverse events profiles. Some of these side effects such as endocrine toxicity can be permanent, and, rarely, life-threatening due to myocarditis, pneumonitis, colitis, and neurologic events [11].

Immune escape is one of the initial steps of metastasis and is crucial for diverse steps of metastasis including the onset of the tumor, dissemination, and survival in the bloodstream, and eventually reaching new organs. The regulation of immune checkpoints in the tumor microenvironment plays a monumental role in the tumor dissemination and immune escape. TAMs residing in the tumor microenvironment promotes the expression of PDL1 which drives to the suppression of cytotoxic T lymphocytes (CTLs) in the tumor microenvironment, this is a mechanism employed frequently to induce metastasis [12]. Moreover, cytokine formation plays substantial roles during metastasis through the stimulation of immune checkpoints. LAG-3+ pDCs possess high potentials in producing IL-6, which suppresses the immune system via STAT3 signaling and leads to melanoma metastasis [13]. IL-8 is one of the cytokines that its formation is triggered by inhibitory immune checkpoints such as B7-H3 [14] and CD73 [15]. IL-8 provokes the expression of integrin αM on neutrophils that can interact with intercellular adhesion molecule 1 (ICAM1) expressed by tumor cells and results in the adherence of tumor cells to the liver sinusoids and the formation of metastatic foci [16]. The goal of this literature review is to compare two categories of immune checkpoints target and their associated immune-landscape impacting cancer progression.

### The potentials of immune checkpoints during the cancer dissemination

Increased expression of inhibitory immune checkpoints has been reported frequently and this increase in the tumor microenvironment stimulates metastasis through varied mechanisms. For instance, PD-1 expression triggers metastasis through the formation of interferon-gamma (IFN-γ), tumor necrosis factor‐alpha (TNF-α), and IL-8 and targeting the JAK2/Stat3/Slug signaling pathway in pancreatic ductal adenocarcinoma (PDAC), melanoma, urinary bladder cancer (UBC), and hepatocellular carcinoma (HCC), respectively [17–20]. CD73 provokes metastasis in cervical cancer and colorectal cancer (CRC) via VEGF/Akt pathway [21] and the MAPK/ERK signaling pathway [22], respectively. CD73 expression facilitates the adherence of metastatic cells to the ECM of the new organ through the LFA1 clustering and adenosine formation [23]. CD73 promotes metastasis of breast cancer via the expression of EGFR and IL-8 [15], while, CD73 blockade restrains melanoma metastasis through the formation of IL-1β and TNF-α [24].

PI3Kγ induces metastasis through increasing the formation of PDGF-BB and improving the expression of MMP-9, uPA, VEGF, HIF-1α, and HIF-2α in PDAC [25] and melanoma [26], respectively. Tim-3 follows diverse approaches to induce metastasis in different kinds of tumors and plays a critical role during the initial steps of metastasis. Tim-3 promotes tumor cell infiltration and diffusion through EMT stimulation [27], GATA3 inhibition [28], and survival in the bloodstream via anoikis prevention [27]. Moreover, Tim-3 expression induces metastasis of HCC, ductal breast carcinoma, prostate cancer, and LAC through promoting macrophages into the M2-like phenotype [29], IL-6-STAT3 pathway [30], reducing the IFN-γ synthesis of peripheral NK cells [31], and triggering the NF-κB signaling [32], respectively.

B7-H3 expression plays a crucial role during metastasis since the expression of this molecule can encourage metastasis through augmenting IL‐8 formation and Stat3, upregulation of MMP-2, downregulation of TIMP‐1 and TIMP‐2 [33], and upregulation of cyclin D1, Stat3, and p-Stat3 [34]. B7-H3 induces its facilitating effects on metastasis of melanoma [33], HCC [35], and osteosarcoma [36] via potentiation of MMP-9. The expression of costimulatory immune checkpoints demonstrates a decline in the tumors associating with metastasis since the expression of these molecules can restrict metastasis through potentiating the formation of IL17A, IFN-γ, and TNF-α and enhancing the cytotoxicity of CD8+ T cells, NK cells, and macrophage [37–39]. CD40L expression impedes metastasis of CRC through the stimulation of NK cells and CTLs and the hindrance of the suppressive effect of Tregs [40, 41]. The expression GITR‐L also hinders melanoma metastasis via augmenting CTLs [42]. CD27 signaling suppresses metastatic RCC via potentiating CD8+ and CD4+ T cells differentiation and enhancing the expression of HLA-DR and costimulatory markers such as ICOS, 4-1BB, and CD69 [43]. 4-1BB restricts metastasis of TNBC, melanoma, and CRC by potentiating CTL [44], increasing the number of TILs [45], and CD11b+ monocytes [46], respectively.

### Costimulatory immune checkpoints

**Fig. 1:**
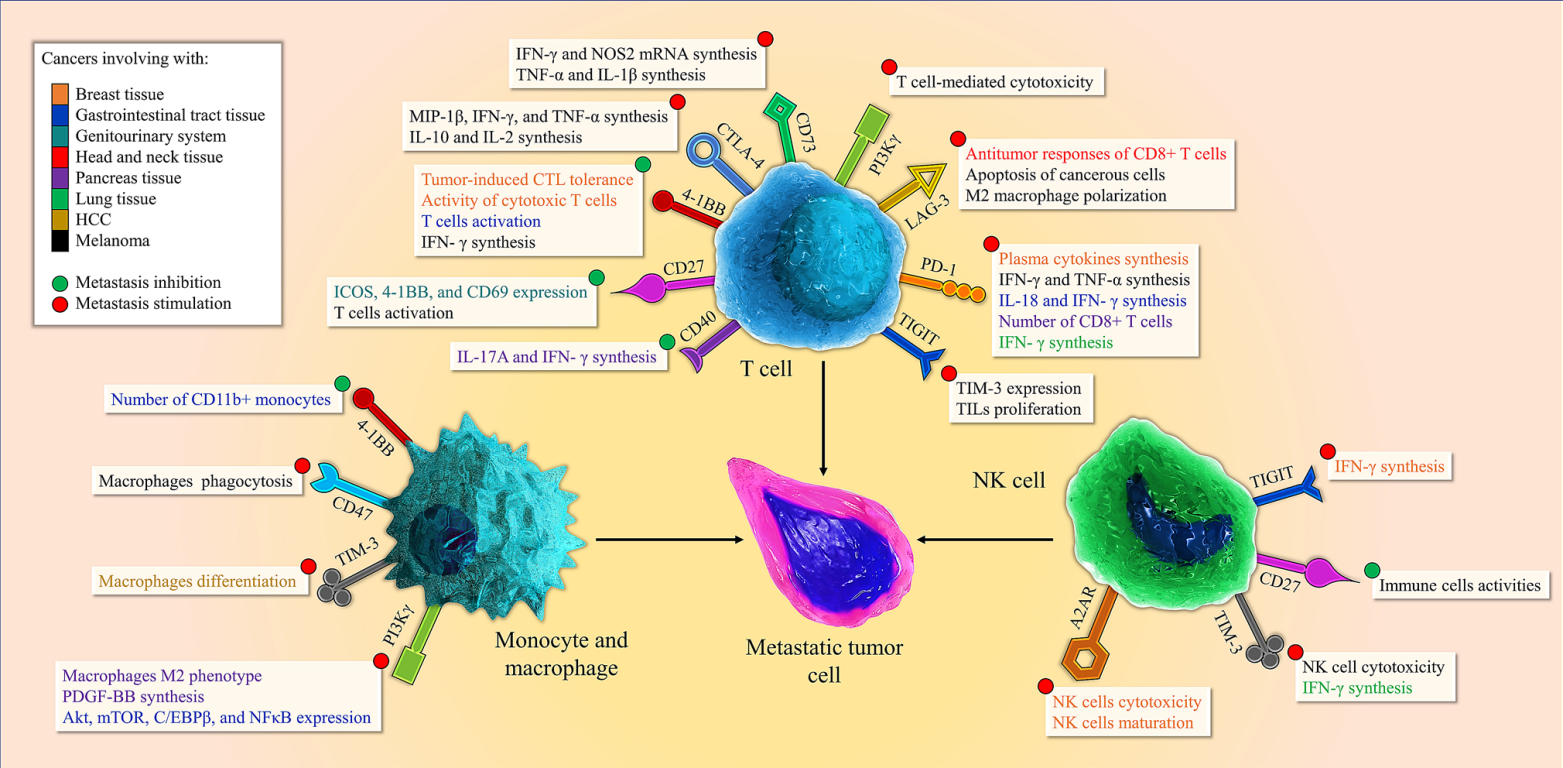
Diverse roles of the immune checkpoint in T cell, NK cell, monocyte, and macrophage, which lead to the stimulation, or inhibition of metastasis. The expression of A2AR, TIM-3, and TIGIT on NK cells plays a stimulating role during metastasis while CD27 impedes metastasis via the regulation of immune cell activities. The expression of inhibitory immune checkpoints including CD47, TIM-3, and PI3Kγ on monocyte and macrophages promotes metastasis. The stimulation of 4-1BB which is a costimulatory immune checkpoint on monocyte can restrict metastasis via increasing the number of CD11b^−^ monocyte. T cells expressing inhibitory immune checkpoints including CTLA-4, CD73, PI3Kγ, LAG-3, PD-1, and TIGIT stimulate metastasis, however, the expression of costimulatory immune checkpoints including 4-1BB, CD27, and CD40 on these cells hinders metastasis

Costimulatory immune checkpoints expression on the immune cells leads to the stimulation and activation of these cells increasing anti-tumor responses (Fig. 1). Tumor cells stimulate tumorigenesis pathways through the inhibition of these costimulatory pathways, which summarized in Table 1.

**Table 1:** Key costimulatory immune checkpoints and their functions in the regulation of cancer metastasis

Tumors	Immune checkpoints	Expressing cells	Kinds of trials	Functions	References
TNBC	4-1BB	T cells	Animal testing	Augmenting the activity of CTL and induction of more differentiated CD8^+^ T cell gene profile	[47]
Breast cancer	4-1BB	T cells	Animal testing	Reversing the tumor-induced CTL tolerance, which will lead to augmented activity of CTL	[48]
Melanoma	4-1BB	T cells	Animal testing	Increasing the population of TIL including CD4^+^ T cells, CD8^+^ IFN‐γ^+^ T cells, and CD11b^+^ TIL in lung tumor masses	[49]
CRC	4-1BB	Monocytes and splenic DCs	Animal testing	Expanding the number CD11b^+^ monocytes or CD11c^+^ splenic DCs	[46]
CRC	4-1BB	T cells	Animal testing	Activating T cells	[50]
RCC	CD27	T cells	Clinical trial	Potentiating the immune responses such as CD8^+^ T cells and CD4^+^ T cells differentiation	[43]
Melanoma	CD27	T cells and NK cells	Animal testing	Augmenting the activities of immune cells such as CD8^+^ and CD4^+^ T cells residing in the tumor microenvironment, FoxP3-expressing CD4^+^ T, and CD3 − NK1.1^+^ NK cells	[51]
SCC	OX40	T cells	Clinical trial	Potentiating T cells responses in the presence of CD3^+^CD4^+^CD25^high^CD127^low^ Treg population	[52]
Melanoma	CD40	Melanoma cells	Animal testing	Enhancing the formation of CD8^+^ T cells cytokines including, IFN-γ, TNF-α, IL-6, IL-13, and GM-CSF	[38]
CRC	CD40	RCN9 cells	Animal testing	Inducing the antitumor responses of Th1 and hindering of the suppressive effect of Tregs	[41]
Breast cancer	CD40	Endothelial progenitor cells	Animal testing	Promoting the production of TNF‐α and INF‐γ and caspase 3/7 activity	[53]
Melanoma	GITR	DCs	Animal testing	Improving the responses of CTL	[54]
Melanoma	GITR	DCs	Animal testing	Enhancing the induction of melanoma tumor‐associated Ag‐specific CTL activity	[42]

*TNBC* triple-negative breast cancer; *CTL* cytotoxic T lymphocyte; *CRC* colorectal cancer; *DCs* dendritic cells; *RCC* renal cell carcinoma; *SCC* squamous cell carcinoma; *IFN-γ* interferon-gamma; *TNF‐α* tumor necrosis factor‐alpha

#### 4-1BB

The 4-1BB expression can inhibit metastatic triple-negative breast cancer (TNBC) cells through augmenting the activity of cytotoxic T cell and induction of a more differentiated CD8^+^ T cell gene profile [50]. 4-1BB stimulation restrains the metastasis of breast cancer through the reversion of tumor-induced CTL tolerance, which will lead to augmented activity of CTL [55]. It has been indicated that induced expression of 4-1BB by the administration of agonistic anti‐4‐1BB mAb restricts the metastasis of B16F10 melanoma cells to the lungs through increasing the population of tumor‐infiltrating lymphocytes (TIL) including CD4^+^ T cells, CD8^+^ T cells, and CD11b^+^ TIL in the lung tumor masses. Moreover, 4-1BB expression increases the number of CD8^+^ IFN‐γ^+^ T cell and enhances the expression of MHC class Ι and II antigens on B16F10 cells in response to increased production of IFN-γ [45]. The usage of agonistic anti-4-1BB mAb also suppresses the metastasis of CT26 CRC cells to the liver through expanding the number of CD11b^+^ monocytes or CD11c^+^ splenic dendritic cells (DCs) [46]. 4-1BB cannot induce long-term survival, while, interleukin 12 (IL-12) can induce long-term survival in 20–30% of liver metastasis models and cases of concurrent use of IL-12 and 4-1BBL, long-term survival will increase by 62% [49].

#### CD27

Augmenting the signaling pathway of CD27 potentiates the immune responses such as CD8^+^ and CD4^+^ T cells differentiation and enhances the expression of HLA-DR and the activation markers on CD4^+^ T cells and restrains metastatic renal cell carcinoma (RCC) [43]. Induced expression of CD27 can impede lung metastasis of melanoma. Enhanced CD27 expression on immune cells such as CD8^+^ and CD4^+^ T cells residing in the tumor microenvironment, FoxP3-expressing CD4^+^ T, and CD3^−^NK1.1^+^ natural killer (NK) cells augments the activities of these cells [51].

#### OX40

OX40 expression increases the possibility of lymph node metastasis from 78.2 to 92.3% in invasive ductal carcinoma of the breast [56]. Furthermore, increased expression of OX40 on TILs has been reported in metastatic cutaneous squamous cell carcinoma (SCC). There is a large number of regulatory T cells (Tregs) in the tumor microenvironment of SCC, which induce the metastasis through the inhibition of antitumor responses of T cells. Interestingly, OX40 expression can potentiate T cells responses while its expression occurs in the presence of CD3^+^CD4^+^CD25^high^CD127^low^ Treg population [52]. It has been indicated that OX40L:Ig administration for the treatment of mice suffering from the tumor-induced by the injection of 4T1 breast cancer cells possessing high potentials for metastasis can inhibit the tumor and improves the survival [57].

#### CD28

CD28‐mediated costimulatory pathways play a significant role during the differentiation of functional tumor‐specific CD8^+^ T‐effector cells and CD28 inexpression in patients suffering from melanoma will result in pulmonary metastases [58]. CD28 expression is declined on metastatic melanoma cells while its expression shows an increase in CD4^+^ lymphocytes that are migrating toward tumors [59]. CD28 expression is improved on CD4^+^ and CD8^+^ T cell surrounding metastatic melanoma cells and in the expression of CD28 on T lymphocytes circulating in peripheral blood of patients suffering from metastatic breast cancer is associated with poor prognosis [60].

#### CD40

CD40 expression on melanoma cells stimulates the formation of CD8 T cell cytokines including IL-13, IL-6, TNF-α, and granulocyte/macrophage colony-stimulating factor (GM-CSF) and impedes brain metastasis which is common among melanoma patients [38]. The systemic injection of endothelial progenitor cells derived from human induced pluripotent stem cells expressing CD40 by the use of baculovirus encoding CD40 ligand inhibits metastasis and induces prolonged survival through the formation of tumor necrosis factor-α (TNF-α) and IFN-γ in the 4T1 breast cancer lung metastasis model [53]. 93 percent of lung tumors expressing CD40 have nodal or systemic metastasis during the initial diagnosis of cancer [61]. CD40 expression on esophageal squamous cell carcinoma (ESCC) leads to the progression of cancer and metastasis to lymph nodes [62]. It has been demonstrated that the administration of the anti-CD40 antibody in female BALB/c mice suffering from mesothelioma inhibits metastasis and improves their survival [63].

Nitric oxide (NO) production induced by IL-2/α-CD40 combination treatment results in the increased and decreased expression of E-cadherin and matrix metalloproteinase (MMP), respectively in the RCC microenvironment. Decreased expression of E-cadherin is involved in the increased probability of metastasis. IL-2/α-CD40 combination therapy has enough potential to induce the IFN-α– and NO-dependent reduction of MMP9 expression in the tumor microenvironment and diminishes the probability of metastasis development to the lung [64]. It has been demonstrated that induced expression of CD40L by adenovirus vector-expressing mouse CD40L hampers metastasis and improves survival in rat metastatic liver cancer cells. Anti-tumor effects of CD40 have attributed to its ability in the induction of Th1 anti-tumor responses and the impeding the suppressive effect of T-regulatory cells [41].

#### GITR

GITR (glucocorticoid-induced tumor necrosis factor receptor) expression on CD8^+^ cells of patients suffering from advanced and metastatic breast cancer is declined in comparison to healthy people while its expression demonstrates a significant increase in CD4^+^ cells [65]. The concurrent administration of Sunitinib which is a multitargeted tyrosine kinase inhibitor with the anti (α)‐GITR agonist can restrict the liver metastasis of metastatic RCC through the induction of activation, proliferation, and enhanced cytotoxicity of CD8^+^ T cells, NK cells, macrophage, and DCs. The examination of isolated CD8^+^ T cells and NK cells from Sunitinib/α‐GITR‐treated mice indicated that these cells encountered increased production of IFN‐γ after PMA/ionomycin stimulation [39]. In this experiment, the tumor was induced to C57BL/6 (H-2^b^) mice by the injection of B16-F10.9 cells, which are an extremely metastatic clone of the B16-F10 melanoma cell line. The injection of DCs transfected with either anti‐GITR mAb mRNA or mRNA encoding soluble GITR‐L to these mice resulted in the improved responses of CTL through the stimulation of GITR pathway and eventually restrained the metastasis to the lungs [54]. Moreover, the injection of DCs transfected with mRNA encoding soluble GITR‐L fusion protein inhibits metastatic melanoma through enhanced induction of melanoma tumor‐associated Ag‐specific CTL activity in C57BL/6 mice implanted with B16/F10.9 cells [42].

### Inhibitory immune checkpoints

**Fig. 2:**
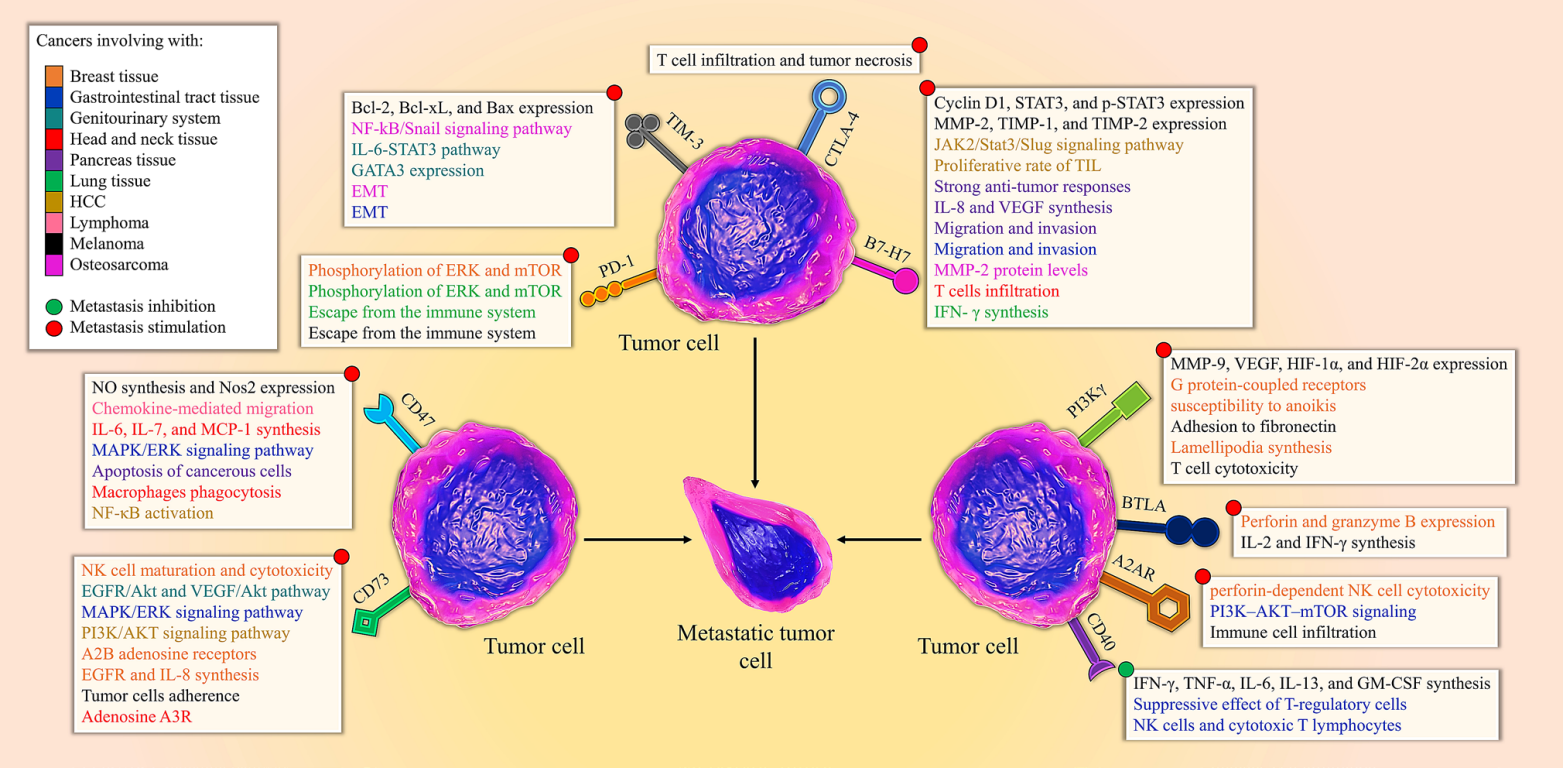
Diverse roles of the immune checkpoint in tumor cells that drive to the stimulation or inhibition of metastasis. Inhibitory immune checkpoints including PD-1, B7-H7, TIM-3, PI3Kγ, CD73, and CD 47 play monumental roles during metastasis; these immune checkpoints employ diverse mechanisms such as IL-8 formation, EMT stimulation, and T cell deterioration. B7-H7 and CD73 induce their promoting role through IL-8 formation during metastasis and the common mechanism of CD73 and A2AR is PI3K/AKT signaling pathway to induce metastasis. The expression of inhibitory immune checkpoints such as CTLA-4, BTLA, and A2AR on tumor cells can promote metastasis while, CD40 expression which is a costimulatory immune checkpoint cripple metastasis via affecting the NK and T cells functions and the synthesis of cytokines

Inhibitory immune checkpoints pathways restrict the activation of T cells and the duration of immune responses and regulate the inflammation, toleration, and homeostasis by employing varied processes (Fig. 2) [66]. Tumors can cripple the immune system by hijacking inhibitory immune checkpoints, which summarized in Table 2.

**Table 2:** Key inhibitory immune checkpoints and their functions in the regulation of cancer metastasis

Tumors	Immune checkpoints	Expressing cells	Kinds of trials	Functions	References
Melanoma	PD-1	T cells	Clinical trial	Declining the production of IFN-γ	[17]
PDAC	PD-1	T cells	Clinical trial	Diminishing the formation of IFN-γ	[67]
Melanoma	PD-1	T cells	Clinical trial	Diminishing the number of IFN-γ and TNF-α producing NY-ESO-1–specific CD8^+^ T cells	[18]
TNBC and prostate cancer	PD-1	MDA-MB-231, 4T1, and DU145 cells	Animal testing	Augmenting the tumor cells against chemotherapy and improving the proliferative potentials of tumor cells through triggering the phosphorylation of ERK and mTOR	[68]
NSCLC and melanoma	PD-1	T cells	Clinical trial	Diminishing the number of CD8^+^ T cells	[69]
NSCLS and melanoma	PD-1	NSCLS and melanoma cells	Clinical trial	Escaping from the immune system	[70]
UBC	PD-1	T cells	Clinical trial	Disturbing the formation of IL-18 and IFN- γ and subsequently enervating the proliferation of naive and memory CD8^+^ T cells	[20]
Melanoma	B7‐H3	MDA-MB-435, FEMX-I, and MDA-MB-435 cells	Animal testing	Potentiating the Stat3 phosphorylation level and IL‐8 formation, upregulating the MMP‐2, and downregulating the TIMP‐1 and TIMP‐2	[33]
Melanoma	B7‐H3	M14 cells	Animal testing	Upregulating the cyclin D1, STAT3, and p-STAT3	[34]
HCC	B7‐H3	HCC cells	Clinical trial	Increasing the activity of MMP-2 and MMP-9 and targeting the EMT by the activation of JAK2/Stat3/Slug signaling pathway	[19]
NSCLC	B7‐H3	NSCLC cells	Clinical trial	Hindering the proliferation and IFN-γ secretion of T cells	[71]
Pancreatic cancer	B7‐H3	Pancreatic cancer cells	Clinical trial	Impeding CD8^+^ T-cell infiltration into tumors and strong anti-tumor responses	[72]
Osteosarcoma	B7‐H3	Osteosarcoma cells	Clinical trial	Diminishing the density of infiltrating CD8^+^ T lymphocytes and improving the MMP-2 protein levels	[36]
HCC	B7‐H3	HCC cells	Clinical trial	Declining the proliferative rate and IFN- γ production of CD4^+^ and CD8^+^ TILs	[73]
Pancreatic cancer and GAC	B7‐H3	Pancreatic cancer and GAC cells	Clinical trial	Increasing the migration and invasion	[74, 75]
Pancreatic cancer	B7‐H3	Aspc-1, Bxpc-3, Sw1990, and Panc-1 cells	Animal testing	Activating the NF-κB signaling through TLR4 upregulation, which would be followed by IL-8 and VEGF expressions	[14]
HNSCC	B7‐H3	HNSCC cells	Clinical trial	Diminishing the numbers of tumor infiltrating CD8^+^ T-cells	[76]
Melanoma	CTLA-4	T cells	Clinical trial	Attenuating the production of MIP-1β, IFN-γ, and TNF-α	[77]
Melanoma	CTLA-4	Melanoma cells	Clinical trial	Hindering the extensive tumor necrosis and CD8^+^ T cell infiltration	[78]
Melanoma	CTLA-4	T cells	Clinical trial	Enhancing the number of T‐regulatory cells and IL‐10 production and diminishing IL‐2 production by activated T cells	[79]
HCC	Tim-3	Macrophages	Animal testing	Inducing the differentiation of macrophages into the M2-like phenotype	[29]
Prostate cancer	Tim-3	Prostate cancer cells	Clinical trial	Stimulating the IL-6-STAT3 pathway	[31]
LAC	Tim-3	NK cells	Clinical trial	Diminishing the cytotoxicity and IFN-γ production of peripheral NK cells	[32]
CCRCC	Tim-3	786-O and Caki-2 cells	Animal testing	Inhibiting the GATA3 expression and stimulating the migration and invasion of CCRCC cells	[80]
Melanoma	Tim-3	NK cells	Clinical trial	Diminishing the NK cell cytotoxicity and inducing the functional exhaustion of T cells	[81]
Melanoma	Tim-3	B16 cells	Animal testing	Triggering the NF-κB signaling pathway and augmenting the proliferation and resistance to apoptosis through upregulation of Bcl-2 and Bcl-xL and downregulation of Bax	[82]
ESCC	Tim-3	Eca109 and TE‑1 cells	Animal testing	Stimulating the EMT	[83]
Osteosarcoma	Tim-3	Osteosarcoma cells	Clinical trial	Stimulating the EMT	[84]
Osteosarcoma	Tim-3	Osteosarcoma cells	Animal testing	Stimulating the EMT and activating the NF-kB/Snail signaling pathway	[27]
Cervical cancer	CD73	Hela and SiHa cells	Animal testing	Augmenting the EGFR/Akt and VEGF/Akt pathway	[21]
CRC	CD73	HCT8 and RKO cells	Animal testing	Activating the MAPK/ERK signaling pathway	[22]
Breast cancer	CD73	4T1.2 cells	Animal testing	Producing the extracellular adenosine and activates A2B adenosine receptors	[85]
HCC	CD73	Hepatocellular carcinoma cells	Clinical trial	Activating the PI3K/AKT signaling by inducing Rap1-mediated membrane localization of P110β	[86]
Breast cancer	CD73	4T1.2 cells and NK cells	Animal testing	Triggering and stimulating the LFA1 clustering and A2A receptors, respectively and promoting the adenosine formation and suppressing NK cell maturation and perforin-mediated NK cell cytotoxicity	[23]
Breast cancer	CD73	T-47D cells	Animal testing	Increasing the expression of EGFR and IL-8 through the improved formation of adenosine	[15]
Melanoma	CD73	T cells	Animal testing	Hindering the expansion of CD11b^+^Gr-1^hi^ myeloid cells and crippling the synthesis of TNF-α and IL-1β	[23]
Melanoma	CD73	T cells	Animal testing	Increasing the number of mannose receptor‐positive macrophages while decreasing the IFN‐γ and NOS2 mRNA production	[87]
Melanoma	CD73	B16F10 cells	Animal testing	Enhancing the adherence of tumor cells to endothelial cells	[88]
HNSCC	CD73	HNSCC cells	Clinical trial	Stimulating the adenosine A3R and activating the signaling of EGF/EGFR	[89]
HCC	CD47	HCC cells	Clinical trial	Promoting CTSS expression via NF‐κB activation	[90]
Glioblastoma	CD47	U87 and CCF-STTG1 cells	Animal testing	Upregulating and downregulating of UHRF1 and p16INK4A, respectively, and improving the expression of inflammatory genes IL-6, IL-7, and MCP-1	[91]
CRC	CD47	DLD-1 cells	Animal testing	Activating the MAPK/ERK signaling pathway	[92]
Leiomyosarcoma	CD47	LMS04 cells	Animal testing	Disturbing the phagocytosis of macrophages	[93]
Melanoma	CD47	Macrophages	Animal testing	Enervating the macrophages phagocytosis	[94]
Lymphoma	CD47	Lymphoma cells	Clinical trial	Enervating the macrophages phagocytosis and promoting the chemokine-mediated migration of lymphoma cells	[95]
PDAC	CD47	PDAC cells	Clinical trial	Enervating the macrophages phagocytosis and impeding the apoptosis of cancerous cells	[96]
Medulloblastoma	CD47	Medulloblastoma cells	Animal testing	Enervating the macrophages phagocytosis	[97]
Melanoma	CD47	Melanoma cells	Clinical trial	Disturbing the abilities of macrophages and the expression of key enzymes involving during NO synthesis and improving the expression of Nos2 mRNA	[98]
Osteosarcoma	CD47	KRIB cells	Animal testing	Enervating the macrophages phagocytosis	[99]
PDAC	PI3Kγ	Macrophages	Animal testing	Suppressing the CD8^+^ cell mobilization, augmenting the formation of PDGF, and inducing the transcription of genes associated with the M2 immunosuppressive macrophages phenotype including Arg1 and Tgfb	[25]
PDAC	PI3Kγ	Macrophages	Animal testing	Enhancing the formation of PDGF-BB	[100]
Gastric cancer	PI3Kγ	Macrophages	Animal testing	Stimulating the Akt, mTOR, and C/EBPβ and inhibiting the NFκB signaling	[101]
Melanoma	PI3Kγ	B16F10 cells	Animal testing	Increasing the expression of MMP-9, uPA, VEGF, HIF-1α, and HIF-2α by TAMs	[26]
Melanoma	PI3Kγ	T cells	Animal testing	Disturbing the polarization of myeloid cells to a less immunosuppressive phenotype, T effector activation, and T cell-mediated cytotoxicity	[102]
Breast cancer	PI3Kγ	Breast cancer cells	Animal testing	Facilitating the function of excessive signaling from G protein-coupled receptors	[103]
Breast cancer	PI3Kγ	MDA-MB-231 cells and MDA-MB-436 cells	Animal testing	Potentiating the synthesis of lamellipodia	[104]
Breast cancer	PI3Kγ	MDA-MB-231 cells	Animal testing	Reducing the susceptibility to anoikis	[105]
Melanoma	PI3Kγ	BLM cells	Animal testing	Reducing the CXCL12-mediated human melanoma cells adhesion to fibronectin and enhancing the invasiveness	[106]
Melanoma	BTLA	B16F1 cells	Animal testing	Crippling the specific cytotoxicity to B16F1 cells and the synthesis of IL-2 and IFN-γ	[107]
Mammary carcinoma	BTLA	Mammary carcinoma cells	Animal testing	Diminishing the number of type I NKT cells and expression of cytotoxic marker genes such as perforin and granzyme B	[108]
HNSCC	LAG-3	T cells	Animal testing	Suppressing the antitumor responses of CD8^+^ T cells	[109]
Melanoma	LAG-3	DCs	Clinical trial	Promoting the synthesis of IL-6 and M2 macrophage polarization	[13]
Melanoma	LAG-3	T cells	Clinical trial	Impeding the apoptosis of MHC II-positive melanoma cells	[110]
Breast cancer	A2AR	AT-3 cells	Animal testing	Inhibiting the perforin-dependent NK cell cytotoxicity	[111]
Gastric cancer	A2AR	MKN45 cells	Animal testing	Enhancing the expression of MMP-2, MMP-7, MMP-9, and MMP-13 and potentiating the PI3K–AKT–mTOR signaling	[112]
Breast cancer	A2AR	NK cells	Animal testing	Suppressing the maturation of NK cells	[113]
Breast cancer and melanoma	A2AR	NK cells	Animal testing	Suppressing the NK cells perforin-mediated cytotoxicity and cytokine synthesis	[23]
Melanoma	A2AR	LWT1 cells	Animal testing	Impeding the immune cell infiltration particularly of CD8^+^ T cells into the tumor microenvironment	[114]
Melanoma	TIGIT	T cells	Clinical trial	Diminishing the proliferation of TILs	[18]
Melanoma	TIGIT	Tregs	Animal testing	Potentiating the expression of the co-inhibitory receptor TIM-3 in tumor tissue and suppresses the immune system	[115]
Breast cancer	TIGIT	NK cells	Animal testing	Crippling the NK cells cytotoxic activities and IFN-γ synthesis	[116]

*PDAC* pancreatic ductal adenocarcinoma; *PD-1* programmed cell death-1; *IFN-γ* interferon-gamma; *TNF‐α* tumor necrosis factor‐alpha; *TNBC* triple-negative breast cancer; *ERK* extracellular signal-regulated kinase; *mTOR* mammalian target of rapamycin; *NSCLC* non-small-cell lung carcinoma; *HCC* hepatocellular carcinoma; *HNSCC* head and neck squamous cell carcinoma

#### Tim-3

Enhanced expression of T cell immunoglobulin and mucin domain-containing protein 3 (TIM3) potentiates metastasis in HCC via induced differentiation of macrophages into the M2-like phenotype [29]. Tim-3 expression delineates an increase in CD8^+^ T cells during the lymph node metastasis originating from ductal breast carcinoma [30]. Moreover, Tim-3 increased expression is positively correlated with lymph node metastasis of head and neck squamous cell carcinoma (HNSCC) [117].

The Tim-3 expression is improved on CD4^+^ T and CD8^+^ T cells isolated from the blood of patients afflicted by the lymph node, central nervous system, and bone metastasis of prostate cancer [118] and Tim-3 expression is also increased along with pulmonary metastasis stemming from prostate cancer and its expression augments metastasis by the stimulation of IL-6-STAT3 pathway [31]. The Tim-3 expression is enhanced on CD3-CD56^+^ NK cells belonging to the patients suffering from lung adenocarcinoma (LAC) with lymph node metastasis since its expression can lead to the diminished cytotoxicity and IFN-γ synthesis of peripheral NK cells [32]. Furthermore, Tim-3 improved expression on CD4^+^ TILs is associated with lymph node metastasis stemming from non-small-cell lung carcinoma (NSCLC) [119]. TIM-3 ligation promotes metastasis of CCRCC through the inhibition of GATA3 since GATA3 inhibition stimulates the migration and invasion of CCRCC [28]. Moreover, TIM-3/ Galectin-9 ligation diminishes the NK cell cytotoxicity and induces the functional exhaustion of T cells in metastatic melanoma [81]. The engagement of a non-galectin 9 putative receptor on B16 melanoma cells with endothelial cell-expressed Tim-3 triggers the NF-κB signaling pathway in B16 cells. The activated NF-κB signaling augments the proliferation and resistance to apoptosis through upregulating the Bcl-2 and Bcl-xL and downregulating the Bax in these tumor cells and promotes the formation of metastatic nodules in the lung [82].

Increased expression of Tim-3 has been detected along with the lymph node and tumor node metastasis originating from ESCC. EMT is one of the most substantial steps during the metastasis of solid tumors and Tim-3 stimulates the EMT in ESCC and subsequently leads to metastasis [83]. Moreover, Tim-3 enhanced expression is associated with increased expression of EMT biomarkers including Slug, Snail, and Smad in osteosarcoma [84]. Tim-3 expression on MG-63 osteosarcoma cells promotes metastasis through the stimulation of EMT and activating the NF-kB/Snail signaling pathway. One of the molecular characteristics of EMT is the downregulation of E-cadherin, which encourages tumor cell infiltration and diffusion [27].

Detached tumor cells from the basement membrane or extracellular matrix (ECM) enter the bloodstream and move toward anoikis, but if anoikis is ceased, metastasis will be initiated [120]. Tim-3 promotes metastasis of RCC through the potentiation of invasiveness and weakening the anoikis stemming from ECM detachment. Anoikis is a special form of programmed cell death and is induced by disengagement from the surrounding ECM or adjacent cells. Anoikis is one of the most significant features of metastasis [28]. Inversely, it has been depicted that low Tim-3 mRNA levels in the tumor tissue and blood mononuclear cells are significantly correlated with lymph node metastasis and distant metastasis of colorectal cancer [121]. The Tim-3 expression is also increased on NK cells belonging to patients with non-metastatic CRC [122]. Although, Tim-3 expression on the HCT116 and HT-29 cells triggers distant and tumor node metastasis through the promotion of invasiveness and migration [123].

#### CD73

CD73 expression on two human cervical cancer cell lines Hela and SiHa encourage metastasis through the augmentation of EGFR/Akt and VEGF/Akt pathway, which plays a significant role during metastasis [21]. Improved expression of CD73 indicates a strong positive correlation with metastasis stemming from CRC and stimulates metastasis through the activation of the MAPK/ERK signaling pathway [22, 124]. The production of extracellular adenosine by tumor-derived CD73 promotes breast cancer metastasis to the lung through the activation of A2B adenosine receptors since the administration of anti-CD73 mAb can reduce the number of spontaneous lung metastases originating from the injection of breast cancer 4T1.2 cells into the mammary fat pad of female wild-type BALB/c mice [85]. CD73 expression assists metastasis in HCC via activating the PI3K/AKT signaling by inducing Rap1-mediated membrane localization of P110β [86]. CD73 expression encourages lung metastasis of 4T1.2 and tumor cells via triggering the LFA1 clustering and adenosine formation since tumor cells employ these two mechanisms to enhance their attachment to the ECM, which is a crucial factor for promoting lung metastasis. Actually, adenosine formation by CD73 stimulates A2A receptors and suppresses the immune system mechanisms such as NK cell maturation and perforin-mediated NK cell cytotoxicity by the use of this stimulation [23]. Furthermore, CD73 expression on T-47D human breast cancer cells potentiates metastasis of breast cancer by increased expression of the epidermal growth factor receptor (EGFR) and IL-8 through the improved formation of adenosine [15]. CD37 inhibition suppresses lung metastasis originating from melanoma through the induction of expansion of CD11b^+^Gr-^1hi^ myeloid cells and enhancing the synthesis of TNF-α and interleukin 1β (IL-1β) [24].

Lack of CD73 expression on B16F10 cells injected into mice intravenously reduces lung metastasis by 3–4 times. Moreover, CD73 expression on endothelial cells is essential to induce metastasis in a manner independent from immunosuppressive effects [125]. Although, it has been indicated that CD73 expression demonstrates no effects on promoting the metastasis of B16-F10 cells and its expression on host cells especially hematopoietic and endothelial cells have no facilitating effects on the metastatic spread of B16-F10 cells probably because of the ineffective formation of adenosine by the tumor itself [126]. It has been illustrated that the induction of melanoma metastasis declines in mice lacking CD73 dramatically since, among TILs belonging to these mice, the numbers of mannose receptor‐positive macrophages are decreased while IFN‐γ and NOS2 mRNA production is increased [87]. CD73 expression on B16F10 cells enhances their adherence to the endothelial cells and increases metastasis probability since the utilization of AOPCP (adenosineα, β-methylene 5′-diphosphate) which inhibits specific tumor cell-ECM interactions through CD73 was able to decline tumor cells adherence [88].

CD73 expression on HNSCC cells stimulates lymph node metastasis through stimulating the adenosine A3R and activating the signaling of EGF/EGFR [89]. The expression of CD73 is increased on advanced rectal adenocarcinoma cells associating with liver and lymph node metastasis [127]. It has been delineated that there is no difference between the expression of CD73 in NSCLC cells and lymph node metastasis [128]. While CD73 expression demonstrates an increase in metastasis of human CRC [129]. CD73 expression is enhanced in laryngeal lesions and lesions of the oral cavity originating from HNSCC during lymph node metastasis [130]. Moreover, lymph node metastasis stemming from the injection of MDA‐MB‐435 cells into the mammary fat pad of mice is associated with increased expression of CD73 [131]. The expression of this molecule on lymph node metastasis of prostate cancer is increased in comparison to normal lymph nodes [132].

It has been revealed that CD73 expression is improved in patients afflicted by lung metastasis of metastatic melanoma and metastasis originating from gastric carcinoma [133, 134]. Furthermore, the induction of cancer by the injection of MB‐MDA‐231 cells into mice is positively correlated with increased expression of CD73 [135]. Reversely, the expression of this molecule in the peritoneum, omentum, and ovary metastasis of endometrial tumors is associated with a decline since CD73-generated adenosine diminishes metastasis through induction of epithelial integrity. In this survey, it has been determined that CD73-generated adenosine promotes cortical actin polymerization through adenosine A1 receptor induction of a Rho GTPase CDC42-dependent conformational change of the actin-related proteins 2 and 3 actin polymerization complex member N-WASP [136].

#### CTLA-4

Increased cytotoxic T-lymphocyte antigen 4 (CTLA-4) expression has been recorded on CD8^+^ and CD4^+^ TALs isolated from metastatic ovarian cancer microenvironment [137]. It has been indicated that anti-CTLA-4 administration can potentiate the immune responses of NY-ESO-1 antigen-specific B cell and T cells through augmented production of MIP-1β, IFN-γ, and TNF-α in patients suffering from metastatic melanoma [138]. The administration of anti–CTLA-4 does not demonstrate any effects on the restriction of lung metastasis of melanoma, however; concurrent use of this treatment with F10/g-vaccinated mice can result in suppressed lung colonization and eradicated pulmonary metastases via increased infiltration of mononuclear cells [139].

The usage of adjunctive CTLA-4 blockade immediately after primary prostate tumor resection can diminish the rate of metastatic relapse from 44 to 97.4% in the lymph nodes [140]. Moreover, the administration of CTLA-4 blockade can induce anti-tumor responses against CNS metastasis in patients suffering from melanoma [141]. The administration of the CTLA-4 blocking antibody MDX-CTLA-4 decreases blood CA-125 levels by 48%, 2 months after the initiation of treatment while this response is not durable and the second infusion of MDX-CTLA-4 can maintain CA-125 levels for 2 months. MDX-CTLA-4 administration can suppress metastasis to the CNS, lungs, abdomen, and soft tissues through the induction of extensive tumor necrosis and CD8^+^ T cell infiltration in patients suffering from metastatic melanoma, which previously vaccinated with irradiated cancerous cells engineered to form granulocyte–macrophage colony-stimulating factor [78]. The utilization of ipilimumab, which is a CTLA-4 blocker, has demonstrated promising results in fighting against the metastatic tumors especially metastatic melanoma [142]. The administration of ipilimumab can be followed by restrained bone and lung metastasis resulting from metastatic RCC. Ipilimumab utilization also shows substantial effects on the suppression of metastatic castration-resistant prostate cancer and declines prostate-specific antigen levels from 650 ng/ml in the first day of treatment to 0 ng/ml in 84th day of the treatment [143, 144]. Furthermore, the administration of ticilimumab, a human monoclonal antibody against CTLA-4 can create sufficient anti-tumor responses against melanoma metastasizing to the subcutaneous tissues, lymph nodes, and lung through decreasing the number of Tregs and IL‐10 production and elevating IL-2 production by activated T cells [79].

#### PD-1

Programmed cell death-1 (PD-1) expression on CD4^+^ and CD8^+^ TILs is increased during cutaneous metastasis originating from melanoma and this increase leads to declined production of IFN-γ in these cells [17]. A previous study has delineated that inhibited PD-1 expression enhances the percentage of CD8^+^ splenocytes and CD8^+^ TIL and the formation of IFN-γ in patients suffering from metastatic PDAC [67]. Furthermore, PD-1 blockade on IFN-γ– and TNF-producing NY-ESO-1-specific CD8^+^ T cells isolated from peripheral blood mononuclear cells belonging to patients suffering from metastatic melanoma increases the number of these cells and ameliorates the therapeutic process of these patients [18]. The stimulation of PD-1/PD-L1 on MDA-MB-231 and 4T1 tumor cells derives to doxorubicin resistance and on DU145 cells encounters docetaxel resistance, which would be followed by metastasis in all of these tumor cells. The activation of the PD-1/PD-L1 pathway triggers the phosphorylation of ERK and mTOR in MDA-MB-231 cells, potentiates the proliferative potential of tumor cells, and initiates the resistance to chemotherapy [68].

The inhibition of PD-1 engagement by the use of pembrolizumab increases the number of CD8^+^ T cells during liver metastasis resulting from melanoma and the NSCLC while the number of CD8^+^ T cells at the invasive margin is declined dramatically [69]. Surprisingly, PD-1^+^ lymphocytes and the ratios between PD-1^+^ and CD8^+^ lymphocytes have delineated a negative correlation with the progress levels of brain metastasis of melanoma and NSCLS which indicates that brain metastasis escapes from the immune system by increased expression of PD-1 at its initial steps. PD-L1 improved expression demonstrates a strong positive correlation with the abundance of FOXP3-positive lymphocytes [70]. Improved expression of PD-L1 is associated with increased tumor size in sentinel lymph node biopsy of metastatic melanoma [145]. Moreover, PD-L1 expression has been observed in circulating tumor cells isolated from patients suffering from metastatic bladder cancer [146].

Enhanced expression of PD-L1 has been detected on the immune cells isolated from patients suffering from intestinal and peritoneal metastasis originating from metastatic melanoma. Moreover, PD-L2 expression on metastatic melanoma cells, DCs, and histiocytes isolated from patients suffering from metastatic melanoma encounter increased rates. Both PD-L1 and 2 expressions on tumor cells delineate a strong positive correlation with CD3^+^, CD4^+^, CD8^+^, FoxP3^+^ cells [147]. PD-L1 expression is associated with 32% of primary CCRCC patients and 23% of metastatic ones. Thus, PD-L1 expression does not demonstrate significant differences between primary and metastatic CCRCC. Furthermore, the expression of this molecule on tumor-infiltrating mononuclear cells is associated with negligible differences between primary and metastatic conditions of this carcinoma [148]. It has been also revealed that PD-L1 and PD-L2 expressions are increased by 53% and 36% of breast cancer brain metastases (BCBM) respectively. PD-1 expression is detected on TILs isolated from 23% of patients suffering from BCBM and its increased expression is correlated with aging. In this survey, no correlation has been reported between PD-1 expression on TILs and PD-1 ligands in BCBM [149]. Increased expression of PD-L1 is associated with higher WHO tumor grade (grade 3) in metastatic gastroenteropancreatic neuroendocrine tumors [150]. PD-L1 expression in patients suffering from lymph node metastasis of gastric carcinoma demonstrates a significant increase [151]. Furthermore, stronger expression of PD-L1 has been detected in the metastatic samples of CCRCC in comparison to primary samples and its augmented expression on tumor cells and infiltrating lymphocytes of patients suffering from metastatic renal cell carcinoma has been demonstrated [67, 150, 152–156].

#### B7‐H3

B7‐H3 expression is increased in both primary and metastatic melanoma and its impediment declines metastasis to the brain, tibia, columna, lung, and liver, dramatically, through declining the signal transducer and activator of transcription 3 (Stat3) phosphorylation level, reducing IL‐8 formation, downregulation of matrix metalloproteinase-2 (MMP-2), and upregulation of tissue inhibitor of metalloproteinases 1 (TIMP‐1) and TIMP‐2 [33]. It has been demonstrated that B7‐H3 expression is enhanced in metastatic melanoma in comparison to primary melanoma and promotes metastasis through the upregulation of cyclin D1, Stat3, and p-Stat3 [34]. Moreover, increased B7‐H3 expression is associated with increased probability of lymph node metastasis of HCC and B7‐H3 stimulation promotes metastasis through increasing the activity of MMP-2 and MMP-9 and targeting the EMT by the activation of JAK2/Stat3/Slug signaling pathway [19]. It has been illustrated that the expression of B7‐H3 is improved in lymph node metastasis resulting from NSCLC because its expression on NSCLC cells hinders the proliferation and IFN-γ secretion of T cells [71]. B7‐H3 expression on pancreatic cancer cells plays a significant role in promoting lymph node metastasis since its inhibition leads to the potentiation of CD8^+^ T cell infiltration into the tumors and induces strong antitumor responses [72]. Increased B7-H3 expression is concurrent with pulmonary metastasis of osteosarcoma as its expression is associated with the diminished density of infiltrating CD8^+^ T lymphocytes and improved MMP-2 protein levels. MMP-2 plays a substantial role in osteosarcoma invasiveness [36]. B7-H3 expression depicts a positive correlation with the progression of tumor-node-metastasis resulting from HCC and its expression stimulates metastasis through declining the proliferative rate and IFN- γ synthesis of CD4^+^ and CD8^+^ TILs [73].

Increased expression of B7-H3 promotes metastasis of pancreatic cancer and gastric adenocarcinoma (GAC) through increasing the migration and invasion [74, 75, 157]. Human pancreatic cancer cells expressing B7-H3 can produce soluble B7-H3 and its expression would be increased along with the movement of these tumor cells toward metastasis. Soluble B7-H3 activates NF-κB signaling through TLR4 upregulation, which would be followed by IL-8, and vascular endothelial growth factor (VEGF) expression and, eventually, leads to metastasis. IL-8 and VEGF expressions play important roles during the induction of metastasis of pancreatic cancer [14]. Furthermore, the expression of B7‐H3 demonstrates an increase during metastasis to the cervical nodes, celiac nodes, and lymph nodes resulting from human pancreatic cancer [158]. The expression of this molecule is ameliorated during lymph node and distant metastasis resulting from CRC [159]. Although, its expression shows no difference during lymph node metastasis of gastric carcinoma [157]. It has been reported that the patients afflicted by distant metastasis of HNSCC possess high levels of B7-H3 expression and its expression is associated with diminished numbers of tumor-infiltrating CD8^+^ T cells [76]. Moreover, nodal and distant metastasis demonstrate a positive correlation with the levels of soluble B7-H3 circulating in patients with NSCLC [160].

#### CD47

CD47 expression demonstrates an increase in patients afflicted by lymph node metastasis originating from ESCC and ovarian cancer [99, 161, 162]. CD47 expression is also positively correlated with lymph node metastasis of luminal-type breast cancer [162]. Furthermore, the expression of CD47 is increased during the lymph node metastasis of invasive CRC [163].

HCC cells expressing CD47 are stimulated to move toward metastasis. Increased probability of tumor node metastasis is associated with the enhanced serum levels of cathepsin S (CTSS) that possess a substantial role during the invasiveness of HCC and CD47 promotes CTSS expression via NF‐κB activation [90]. CD47 engagement encourages metastasis of astrocytoma cell line U87 and CCF-STTG1 through the upregulation and downregulation of UHRF1 and p16^INK4A^ respectively. Moreover, CD47 interaction activates NF-κB transactivation and subsequently, improves the expression of inflammatory genes IL-6, IL-7, and MCP-1 and leads to metastasis [91]. CD47 expression stimulates the proliferation and metastasis of colorectal adenocarcinoma cell DLD-1 through the activation of the MAPK/ERK signaling pathway [92].

The expression of CD47 is enhanced along with the development of leiomyosarcoma cells toward metastasis. CD47 blockade by the use of anti-CD47 (B6H12) on the tumor cells potentiates the phagocytosis of these cells by macrophages and this blockade diminishes lung metastasis of leiomyosarcoma LMS04 cells by 70 times [93]. The employment of anti-CD47 siRNA delineates that CD47 blockade can restrain lung metastasis stemming from melanoma since the lack of CD47 expression on macrophages augments these immune cells phagocytosis and its expression is increased during metastasis development [94].

CD47 blockade declines lymphoma metastasis to the brain, pituitary gland, nasal cavity, bone marrow, pancreas, kidney, and liver, dramatically, through augmenting the phagocytosis of macrophages since CD47 expression depicts an increase in metastatic and disseminated lymphoma in comparison to primary lesions. CD47 promotes chemokine-mediated migration of lymphoma cells and by its blockade, it has been demonstrated that this molecule possesses a notable role in the migration of these cells toward known lymphoma chemo attractants SDF-1α and CXCL13 [95]. CD47 expression is enhanced in primary PDAC and its metastasis and its blockade restricts metastasis through potentiated phagocytosis of pancreatic cancer stem cells by macrophages and induced apoptosis of cancerous cells [96]. The expression of CD47 is improved in metastatic regions of medulloblastoma in comparison to the primary tumor. The employment of a humanized anti-CD47 antibody Hu5F9-G4 diminishes the metastasis in the forebrain and the spine originating from medulloblastoma, notably, via augmenting the phagocytosis of macrophages [97]. CD47 increased expression is positively correlated with the movement of melanoma cells toward metastasis and its blockade suppresses the metastasis through enhancing the abilities of macrophages and the number of differentiated macrophages (by 50%) in the pulmonary sites of metastasis, declining the expression of Nos2 mRNA, and stimulating the expression of key enzymes involving during NO synthesis [98]. Moreover, CD47 expression demonstrates an improvement in osteosarcoma metastasis and the utilization of Anti-CD47 Abs eliminates spontaneous metastasis of KRIB osteosarcoma cells via the potentiation of macrophages phagocytosis [99].

#### PI3Kγ

Phosphatidylinositol 3-kinase-gamma (PI3Kγ) expression on macrophages promotes metastasis of PDAC through the suppression of CD8^+^ cell mobilization into PDACs tissue, the augmented formation of PDGF-BB by macrophages, and the induction of transcription of genes associated with the M2 immunosuppressive macrophages phenotype in PDACs including immunosuppressive factors Arg1 and Tgfb [25]. Furthermore, PI3Kγ expression on macrophages triggers metastasis of PDAC through the enhanced formation of PDGF-BB [100]. PI3Kγ expression on macrophages suppresses the immune responses during the growth of gastric cancer through the stimulation of Akt, mTOR, and C/EBPβ and inhibition of NFκB and eventually increases the numbers of metastatic nodules in the lung [101]. PI3Kγ expression on B16F10 melanoma cells promotes metastasis to the lungs of mice suffering from melanoma caused via the injection of B16F10 cells to their tail veins through increasing the expression of MMP-9, uPA, VEGF, HIF-1α, and HIF-2α by tumor-associated macrophages (TAMs) [26]. It has been delineated that IPI-549 use which is a PI3Kγ inhibitor can reduce lung metastasis resulting from the injection of B16-F10 cells into C57BL/6 J mice since PI3Kγ inhibition can stimulate the polarization of myeloid cells to a less immunosuppressive phenotype and potentiate T effector activation and T cell-mediated cytotoxicity [102, 164].

PI3Kγ expression is increased on metastatic breast cancer MDA-MB-231 and MDA-MB-436 cells and this increase promotes metastasis to the regional lymph node via potentiating the synthesis of lamellipodia [104]. The inhibition of PI3Kγ expression on MDA-MB-231 cells also restricts the metastasis of these tumor cells by increasing the susceptibility to anoikis. Moreover, reduced expression of PI3Kγ can be followed by restrained spontaneous and experimental metastasis in the mouse 4T1 model of breast cancer [105]. PI3Kγ encourages metastasis through reducing the CXCL12-mediated human melanoma cells adhesion to fibronectin and enhancing the invasiveness [106].

#### A2AR

Adenosine A2A receptor (A2AR) delineates an increase in RCC patients who were afflicted by visceral metastases (80%) and hepatic metastases (20%) [165]. A2aR expression is enhanced on metastatic gastric cancer MKN45 cells and adenosine interaction with adenosine receptor A2a provokes the invasiveness and migration of these cells and eventually leads to metastasis through augmenting the PI3K–AKT–mTOR signaling and the expression of MMP-2, MMP-7, MMP-9, and MMP-13 [112]. A2AR stimulation potentiates CD73^+^ breast cancer metastasis via the inhibition of perforin-dependent NK cytotoxicity [111]. Furthermore, A2AR engagement on NK cells promotes lung metastasis through augmenting the immunosuppressive responses such as the inhibition of cytokine synthesis, NK cell maturation, and perforin-mediated NK cell cytotoxicity in patients afflicted by melanoma and breast cancer [23]. It has been indicated that the hindrance of A2AR signaling can restrain lung metastasis through improved immune cell infiltration, particularly CD8^+^ T cells into the tumor microenvironment in an SM1WT1 BRAF-mutated melanoma tumor model [166]. A2AR blockade by the use of PBF-509 can also suppress metastasis to the lungs belonging to mouse models suffering from melanoma and fibrosarcoma caused by intravenous injection of MCA205 and B16F10 cells. Both cell lines possessed CD79 expression [24].

#### BTLA

Increased expression of B- and T-lymphocyte attenuator (BTLA) is detected on gastric cancer cells, which metastasize to the lymph nodes [167]. BTLA expression is also enhanced on B-Cell Lymphoma cells metastasizing to the CNS [168]. BTLA expression delineates an increase in B16F1 cells injected into mice via the tail vein and induces pulmonary metastasis. These tumor cells escape from the immune system by the use of BTLA–HVEM pathway since impeding the signaling of BTLA–HVEM in the cell culture of naive mice splenocytes with B16F1 cells ameliorates specific cytotoxicity to B16F1 cells and the synthesis of IL-2 and IFN-γ [107]. It has been illustrated that by the use of mice expressing the polyomavirus middle T oncoprotein under the mouse mammary tumor virus promoter in a C57BL/6 background, BTLA blockade on mammary carcinoma cells diminishes lung metastasis [108]. Also, BTLA blockade improves the number of types I NKT cells and the expression of cytotoxic marker genes such as perforin and granzyme B [108].

#### LAG-3

Lymphocyte-activation gene 3 (LAG-3) expression is increased on human plasmacytoid DCs isolated from melanoma metastasizing to the lymph node and skin. LAG-3^+^ pDCs possess tight contacts with melanoma cells and form IL-6 actively. IL-6 induces immunosuppressive responses through STAT3 signaling and IL-6 synthesized by plasmacytoid DCs stimulates the monocytes to produce C–C motif chemokine ligand 2 (CCL2) which plays significant roles in the recruitment of myeloid-derived suppressor cells at the tumor site and M2 macrophage polarization and eventually promotes metastasis [13]. Enhanced expression of LAG-3 has been detected on CD4^+^ and CD8^+^ T cells infiltrating into metastatic lymph nodes of patients suffering from melanoma. Furthermore, the numbers of LAG-3^+^ CD4^+^ CD25^+^ FOXP3^+^ T cells infiltrating into metastatic lymph nodes are increased dramatically. The engagement of LAG-3 with MHC II expressing on melanoma cells impedes apoptosis of these tumor cells and provokes metastasis [110].

LAG-3 expression delineates an increase in TILs isolated from metastatic lymph nodes stemming from HNSCC and this increased expression provokes metastasis via making the resistance against the immune system and suppressing the antitumor responses of CD8^+^ T cells [109]. Moreover, the number of extraepithelial and intraepithelial LAG-3^+^ TILs is increased in metastatic lymph nodes originating from NSCLC [169].

#### TIGIT

T cell immunoreceptor with Ig and ITIM domains (TIGIT) expression delineates an increase on tumor cells and antigen-presenting cells (APCs) isolated from the tumor microenvironment belonging to metastatic melanoma patients and this increase on CD8^+^ TILs isolated from metastatic tumor single-cell suspensions from seven patients with advanced melanoma stimulates metastasis through diminishing the proliferation of TILs [18]. TIGIT expression is also enhanced on CD4^+^ and CD8^+^ T cells that infiltrated melanoma tumors in mice afflicted by B16F10 melanoma tumors. Furthermore, TIGIT expression has been detected on Tregs and TIGIT^+^ Tregs suppresses the immune system via improving the expression of the co-inhibitory receptor, TIM-3, in tumor tissue [115]. It has been illustrated that TIGIT inhibition on NK cells suppresses metastatic breast cancer cells, MDA MB-453 through provoking the cytotoxic activities and IFN-γ synthesis of NK cells [116].

#### VISTA

V-domain Ig suppressor of T cell activation (VISTA) expression demonstrates negligible differences in metastatic lymph nodes and primary human OSCC [170]. Increased number of VISTA^+^ lymphocytes has been detected in patients afflicted by metastatic melanoma and VISTA expression is positively correlated with intratumoral nuclear expression of FOXP3^+^ Tregs [171] and melanoma suppresses the immune system responses via upregulating FOXP3^+^ Tregs [172].

## Discussion and conclusion

A thorough review of the literature revealed that recent monumental discoveries in the field of immune checkpoints are showing us a promising future for the treatment of metastasis by the use of costimulatory (Fig. 1) and inhibitory (Fig. 2) immune checkpoints regulation in clinical and animal testing. Metastasis can be considered as the last stage of cancer aggressiveness and according to previous studies, occurring metastasis can reduce the survival of patients afflicted by lung [173] and gastric [174] cancers by 6 months. The expression of inhibitory immune checkpoints that employed by tumor cells for their development, demonstrates an increase in a plethora of tumors and the inhibition of these immune checkpoints can result in promising outcomes to suppress metastasis [30, 163]. On the other hand, diverse studies have revealed that the stimulation of costimulatory immune checkpoints can inhibit metastasis stemming from various cancers such as TNBC, melanoma, and CRC since the inherent nature of these immune checkpoints is to potentiate the immune system [47].

We conclude, based on the current evidence, that there is a lack of cancer treatment efficacy when the primary tumor turns to a metastatic one because metastasis has intricate molecular processes. Fortunately, monumental advancements have been obtained in the field of immune checkpoints during recent years, which shed light upon the treatment of patients suffering from metastatic cancers. The regulation of immune checkpoints in varied tumors can be employed as a novel strategy and weapon to achieve better results for impeding metastasis in the future. Thus, the extension of research to detect the roles of immune checkpoints during metastasis is increasingly needed and strongly suggested.

## Data Availability

Data sharing not applicable to this article as no datasets were generated or analysed during the current study.

## References

[1] Torre LA, Siegel RL, Ward EM, Jemal A (2016). Global cancer incidence and mortality rates and trends–an update. Cancer Epidemiol Biomarkers Prev.

[2] Steeg PS (2016). Targeting metastasis. Nat Rev Cancer.

[3] Mantovani A (2009). Inflaming metastasis. Nature.

[4] Marin-Acevedo JA, Dholaria B, Soyano AE (2018). Next generation of immune checkpoint therapy in cancer: new developments and challenges. J Hematol Oncol.

[5] Pardoll DM (2012). The blockade of immune checkpoints in cancer immunotherapy. Nat Rev Cancer.

[6] Najafi-Hajivar S, Zakeri-Milani P, Mohammadi H (2016). Overview on experimental models of interactions between nanoparticles and the immune system. Biomed Pharmacother.

[7] Greenwald RJ, Freeman GJ, Sharpe AH (2005). THE B7 FAMILY REVISITED. Annu Rev Immunol.

[8] Zou W, Chen L (2008). Inhibitory B7-family molecules in the tumour microenvironment. Nat Rev Immunol.

[9] Sharma P, Allison JP (2015). The future of immune checkpoint therapy. Science (80-).

[10] Topalian SL, Drake CG, Pardoll DM (2015). Immune checkpoint blockade: a common denominator approach to cancer therapy. Cancer Cell.

[11] Postow MA, Sidlow R, Hellmann MD (2018). Immune-related adverse events associated with immune checkpoint blockade. N Engl J Med.

[12] Noy R, Pollard JW (2014). Tumor-associated macrophages: from mechanisms to therapy. Immunity.

[13] Camisaschi C, De Filippo A, Beretta V (2014). Alternative activation of human plasmacytoid dcs in vitro and in melanoma lesions: involvement of LAG-3. J Invest Dermatol.

[14] Xie C, Liu D, Chen Q (2016). Soluble B7–H3 promotes the invasion and metastasis of pancreatic carcinoma cells through the TLR4/NF-κB pathway. Sci Rep.

[15] Zhou P, Zhi X, Zhou T (2007). Overexpression of Ecto-5’-Nucleotidase (CD73) promotes T-47D human breast cancer cells invasion and adhesion to extracellular matrix. Cancer Biol Ther.

[16] Spicer JD, McDonald B, Cools-Lartigue JJ (2012). Neutrophils promote liver metastasis via Mac-1–mediated interactions with circulating tumor cells. Cancer Res.

[17] Chapon M, Randriamampita C, Maubec E (2011). Progressive upregulation of PD-1 in primary and metastatic melanomas associated with blunted TCR signaling in infiltrating T lymphocytes. J Invest Dermatol.

[18] Chauvin J-M, Pagliano O, Fourcade J (2015). TIGIT and PD-1 impair tumor antigen–specific CD8+ T cells in melanoma patients. J Clin Invest.

[19] Kang F, Wang L, Jia H (2015). B7–H3 promotes aggression and invasion of hepatocellular carcinoma by targeting epithelial-to-mesenchymal transition via JAK2/STAT3/Slug signaling pathway. Cancer Cell Int.

[20] Powles T, Eder JP, Fine GD (2014). MPDL3280A (anti-PD-L1) treatment leads to clinical activity in metastatic bladder cancer. Nature.

[21] Gao Z, Wang H, Lin F (2017). CD73 promotes proliferation and migration of human cervical cancer cells independent of its enzyme activity. BMC Cancer.

[22] Liu X, Wu X, Chen Y (2016). Abstract 2938: Role of CD73 in promoting metastasis and resistance to 5-fluorouracil of colorectal cancer. Experimental and Molecular Therapeutics.

[23] Beavis PA, Divisekera U, Paget C (2013). Blockade of A 2A receptors potently suppresses the metastasis of CD73 + tumors. Proc Natl Acad Sci.

[24] Young A, Ngiow SF, Barkauskas DS (2016). Co-inhibition of CD73 and A2AR adenosine signaling improves anti-tumor immune responses. Cancer Cell.

[25] Kaneda MM, Cappello P, Nguyen AV (2016). Macrophage PI3K drives pancreatic ductal adenocarcinoma progression. Cancer Discov.

[26] Joshi S, Singh AR, Zulcic M, Durden DL (2014). A macrophage-dominant PI3K isoform controls hypoxia-induced HIF1α and HIF2α stability and tumor growth, angiogenesis, and metastasis. Mol Cancer Res.

[27] Feng ZM, Guo SM (2016). Tim-3 facilitates osteosarcoma proliferation and metastasis through the NF-κB pathway and epithelial-mesenchymal transition. Genet Mol Res.

[28] Yu M, Lu B, Liu Y (2017). Interference with Tim-3 protein expression attenuates the invasion of clear cell renal cell carcinoma and aggravates anoikis. Mol Med Rep.

[29] Yan W, Liu X, Ma H (2015). Tim-3 fosters HCC development by enhancing TGF-β-mediated alternative activation of macrophages. Gut.

[30] Zhang H, Xiang R, Wu B (2017). T-cell immunoglobulin mucin-3 expression in invasive ductal breast carcinoma: clinicopathological correlations and association with tumor infiltration by cytotoxic lymphocytes. Mol Clin Oncol.

[31] Piao Y-R, Piao L-Z, Zhu L-H (2013). Prognostic value of T Cell immunoglobulin Mucin-3 in prostate cancer. Asian Pac J Cancer Prev.

[32] Xu L, Huang Y, Tan L (2015). Increased Tim-3 expression in peripheral NK cells predicts a poorer prognosis and Tim-3 blockade improves NK cell-mediated cytotoxicity in human lung adenocarcinoma. Int Immunopharmacol.

[33] Tekle C, Nygren MK, Chen Y-W (2012). B7–H3 contributes to the metastatic capacity of melanoma cells by modulation of known metastasis-associated genes. Int J Cancer.

[34] Wang J, Chong KK, Nakamura Y (2013). B7–H3 Associated with tumor progression and epigenetic regulatory activity in cutaneous melanoma. J Invest Dermatol.

[35] Mao Y, Li W, Chen K (2015). B7–H1 and B7–H3 are independent predictors of poor prognosis in patients with non-small cell lung cancer. Oncotarget.

[36] Wang L, Zhang Q, Chen W (2013). B7–H3 is overexpressed in patients suffering osteosarcoma and associated with tumor aggressiveness and metastasis. PLoS ONE.

[37] Beatty GL, Chiorean EG, Fishman MP (2011). CD40 agonists alter tumor stroma and show efficacy against pancreatic carcinoma in mice and humans. Science.

[38] Singh M, Vianden C, Cantwell MJ (2017). Intratumoral CD40 activation and checkpoint blockade induces T cell-mediated eradication of melanoma in the brain. Nat Commun.

[39] Yu N, Fu S, Xu Z (2016). Synergistic antitumor responses by combined GITR activation and sunitinib in metastatic renal cell carcinoma. Int J Cancer.

[40] Iida T, Shiba H, Misawa T (2010). Immunogene therapy against colon cancer metastasis using an adenovirus vector expressing CD40 ligand. Surgery.

[41] Hanyu K, Iida T, Shiba H (2008). Immunogene therapy by adenovirus vector expressing CD40 ligand for metastatic liver cancer in rats. Anticancer Res.

[42] Pruitt SK, Boczkowski D, de Rosa N (2011). Enhancement of anti-tumor immunity through local modulation of CTLA-4 and GITR by dendritic cells. Eur J Immunol.

[43] Burris HA, Infante JR, Ansell SM (2017). Safety and activity of varlilumab, a novel and first-in-class agonist anti-CD27 antibody, in patients with advanced solid tumors. J Clin Oncol.

[44] Martinet O (2000). Immunomodulatory gene therapy with interleukin 12 and 4–1BB ligand: long- term remission of liver metastases in a mouse model. J Natl Cancer Inst.

[45] Pastor F, Kolonias D, McNamara JO, Gilboa E (2011). Targeting 4–1BB costimulation to disseminated tumor lesions with Bi-specific oligonucleotide aptamers. Mol Ther.

[46] Lee H, Park H-J, Sohn H-J (2011). Combinatorial therapy for liver metastatic colon cancer: dendritic cell vaccine and low-dose agonistic Anti-4-1BB antibody co-stimulatory signal. J Surg Res.

[47] Harao M, Gao H, Chen JQ (2015). Abstract Co-stimulation through improves expansion and function of tumor-infiltrating T lymphocytes from primary and metastatic triple-negative breast cancer and inflammatory breast cancer. Poster session abstracts.

[48] Mukherjee P, Tinder TL, Basu GD (2004). Therapeutic efficacy of MUC1-specific cytotoxic T lymphocytes and CD137 co-stimulation in a spontaneous breast cancer model. Breast Dis.

[49] Ju S, Lee S, Kwon T (2005). Immunity to melanoma mediated by 4–1BB is associated with enhanced activity of tumour-infiltrating lymphocytes. Immunol Cell Biol.

[50] Martinet O, Ermekova V, Qiao JQ, Sauter B, Mandeli J, Chen LCS (2000). Immunomodulatory gene therapy with interleukin 12 and 4–1BB ligand: long- term remission of liver metastases in a mouse model. J Natl Cancer Inst.

[51] Roberts DJ, Franklin NA, Kingeter LM (2010). Control of established melanoma by CD27 stimulation is associated with enhanced effector function and persistence, and reduced PD-1 expression of tumor infiltrating CD8+ T cells. J Immunother.

[52] Lai C, August S, Albibas A (2016). OX40 + Regulatory T cells in cutaneous squamous cell carcinoma suppress effector T-cell responses and associate with metastatic potential. Clin Cancer Res.

[53] Purwanti YI, Chen C, Lam DH (2014). Antitumor effects of CD40 ligand-expressing endothelial progenitor cells derived from human induced pluripotent stem cells in a metastatic breast cancer model. Stem Cells Transl Med.

[54] Boczkowski D, Lee J, Pruitt S, Nair S (2009). Dendritic cells engineered to secrete anti-GITR antibodies are effective adjuvants to dendritic cell-based immunotherapy. Cancer Gene Ther.

[55] Chen S-H, Pham-Nguyen KB, Martinet O (2000). Rejection of disseminated metastases of colon carcinoma by synergism of IL-12 gene therapy and 4–1BB costimulation. Mol Ther.

[56] Xie F, Wang Q, Chen Y (2010). Costimulatory molecule OX40/OX40L expression in ductal carcinoma in situ and invasive ductal carcinoma of breast: an immunohistochemistry-based pilot study. Pathol Res Pract.

[57] Morris A, Vetto JT, Ramstad T (2001). Induction of anti-mammary cancer immunity by engaging the ox-40 receptor in vivo. Breast Cancer Res Treat.

[58] Voigt H, Schrama D, Eggert AO (2006). CD28-mediated costimulation impacts on the differentiation of DC vaccination-induced T cell responses. Clin Exp Immunol.

[59] Håkansson A, Håkansson L, Gustafsson B (2002). Biochemotherapy of metastatic malignant melanoma. On down-regulation of CD28. Cancer Immunol Immunother.

[60] Song Q, Ren J, Zhou X (2018). Circulating CD8 + CD28 − suppressor T cells tied to poorer prognosis among metastatic breast cancer patients receiving adoptive T-cell therapy: a cohort study. Cytotherapy.

[61] Sabel MS, Yamada M, Kawaguchi Y (2000). CD40 expression on human lung cancer correlates with metastatic spread. Cancer Immunol Immunother.

[62] Matsumura Y, Hiraoka K, Ishikawa K (2016). CD40 expression in human esophageal squamous cell carcinoma is associated with tumor progression and lymph node metastasis. Anticancer Res.

[63] Khong A, Brown MD, Vivian JB (2013). Agonistic anti-CD40 antibody therapy is effective against postoperative cancer recurrence and metastasis in a murine tumor model. J Immunother.

[64] Weiss JM, Ridnour LA, Back T (2010). Macrophage-dependent nitric oxide expression regulates tumor cell detachment and metastasis after IL-2/anti-CD40 immunotherapy. J Exp Med.

[65] Krausz LT, Fischer-Fodor E, Major ZZ, Fetica B (2012). Gitr-expressing regulatory T-cell subsets are increased in tumor-positive lymph nodes from advanced breast cancer patients as compared to tumor-negative lymph nodes. Int J Immunopathol Pharmacol.

[66] Sharpe AH (2017). Introduction to checkpoint inhibitors and cancer immunotherapy. Immunol Rev.

[67] Soares KC, Rucki AA, Wu AA (2015). PD-1/PD-L1 blockade together with vaccine therapy facilitates effector T-cell infiltration into pancreatic tumors. J Immunother.

[68] Black M, Barsoum IB, Truesdell P (2016). Activation of the PD-1/PD-L1 immune checkpoint confers tumor cell chemoresistance associated with increased metastasis. Oncotarget.

[69] Tumeh PC, Hellmann MD, Hamid O (2017). Liver metastasis and treatment outcome with anti-PD-1 monoclonal antibody in patients with melanoma and NSCLC. Cancer Immunol Res.

[70] Harter PN, Bernatz S, Scholz A (2015). Distribution and prognostic relevance of tumor-infiltrating lymphocytes (TILs) and PD-1/PD-L1 immune checkpoints in human brain metastases. Oncotarget.

[71] Mao Y, Li W, Chen K (2015). B7–H1 and B7–H3 are independent predictors of poor prognosis in patients with non-small cell lung cancer. Oncotarget.

[72] Yamato I, Sho M, Nomi T (2009). Clinical importance of B7–H3 expression in human pancreatic cancer. Br J Cancer.

[73] Sun T-W, Gao Q, Qiu S-J (2012). B7–H3 is expressed in human hepatocellular carcinoma and is associated with tumor aggressiveness and postoperative recurrence. Cancer Immunol Immunother.

[74] Zhao X, Li D-C, Zhu X-G (2013). B7–H3 overexpression in pancreatic cancer promotes tumor progression. Int J Mol Med.

[75] Dai W, Shen G, Qiu J (2014). Aberrant expression of B7–H3 in gastric adenocarcinoma promotes cancer cell metastasis. Oncol Rep.

[76] Katayama A, Takahara M, Kishibe K, Nagato T, Kunibe I, Katada A, Hayashi THY (2011). Expression of B7–H3 in hypopharyngeal squamous cell carcinoma as a predictive indicator for tumor metastasis and prognosis. Int J Oncol.

[77] Yuan J, Gnjatic S, Li H (2008). CTLA-4 blockade enhances polyfunctional NY-ESO-1 specific T cell responses in metastatic melanoma patients with clinical benefit. Proc Natl Acad Sci.

[78] Hodi FS, Mihm MC, Soiffer RJ (2003). Biologic activity of cytotoxic T lymphocyte-associated antigen 4 antibody blockade in previously vaccinated metastatic melanoma and ovarian carcinoma patients. Proc Natl Acad Sci.

[79] Reuben JM, Lee B-N, Li C (2006). Biologic and immunomodulatory events after CTLA-4 blockade with ticilimumab in patients with advanced malignant melanoma. Cancer.

[80] Zheng H, Guo X, Tian Q, Li H (2015). Distinct role of Tim-3 in systemic lupus erythematosus and clear cell renal cell carcinoma. Int J Clin Exp Med.

[81] da Silva IP, Jimenez-Baranda S, Gallois A (2012). Abstract 5410: Tim-3 expression and function in natural killer cells from metastatic melanoma patients. Immunology.

[82] Zhang (2010). Endothelial cell-expressed Tim-3 facilitates metastasis of melanoma cells by activating the NF-κB pathway. Oncol Rep.

[83] Shan B, Man H, Liu J (2016). TIM-3 promotes the metastasis of esophageal squamous cell carcinoma by targeting epithelial-mesenchymal transition via the Akt/GSK-3β/Snail signaling pathway. Oncol Rep.

[84] Shang Y, Li Z, Li H (2013). TIM-3 expression in human osteosarcoma: correlation with the expression of epithelial-mesenchymal transition-specific biomarkers. Oncol Lett.

[85] Stagg J, Divisekera U, McLaughlin N (2010). Anti-CD73 antibody therapy inhibits breast tumor growth and metastasis. Proc Natl Acad Sci.

[86] Ma X-L, Shen M-N, Hu B (2019). CD73 promotes hepatocellular carcinoma progression and metastasis via activating PI3K/AKT signaling by inducing Rap1-mediated membrane localization of P110β and predicts poor prognosis. J Hematol Oncol.

[87] Yegutkin GG, Marttila-Ichihara F, Karikoski M (2011). Altered purinergic signaling in CD73-deficient mice inhibits tumor progression. Eur J Immunol.

[88] Koszałka P, Gołuńska M, Stanisławowski M (2015). CD73 on B16F10 melanoma cells in CD73-deficient mice promotes tumor growth, angiogenesis, neovascularization, macrophage infiltration and metastasis. Int J Biochem Cell Biol.

[89] Ren Z-H, Lin C-Z, Cao W (2016). CD73 is associated with poor prognosis in HNSCC. Oncotarget.

[90] Lee TK-W, Cheung VC-H, Lu P (2014). Blockade of CD47-mediated cathepsin S/protease-activated receptor 2 signaling provides a therapeutic target for hepatocellular carcinoma. Hepatology.

[91] Boukhari A, Alhosin M, Bronner C (2015). CD47 activation-induced UHRF1 over-expression is associated with silencing of tumor suppressor gene p16INK4A in glioblastoma cells. Anticancer Res.

[92] Hu T, Chen Y, Zhou C (2017). Abstract 4866: CD47 promotes metastasis and proliferation of colorectal cancer via MAPK/ERK pathway. Tumor Biology.

[93] Edris B, Weiskopf K, Volkmer AK (2012). Antibody therapy targeting the CD47 protein is effective in a model of aggressive metastatic leiomyosarcoma. Proc Natl Acad Sci.

[94] Wang Y, Xu Z, Guo S (2013). Intravenous delivery of siRNA targeting CD47 effectively inhibits melanoma tumor growth and lung metastasis. Mol Ther.

[95] Chao MP, Tang C, Pachynski RK (2011). Extranodal dissemination of non-Hodgkin lymphoma requires CD47 and is inhibited by anti-CD47 antibody therapy. Blood.

[96] Cioffi M, Trabulo S, Hidalgo M (2015). Inhibition of CD47 effectively targets pancreatic cancer stem cells via dual mechanisms. Clin Cancer Res.

[97] Gholamin S, Mitra SS, Feroze AH (2017). Disrupting the CD47-SIRPα anti-phagocytic axis by a humanized anti-CD47 antibody is an efficacious treatment for malignant pediatric brain tumors. Sci Transl Med.

[98] Ngo M, Han A, Lakatos A (2016). Antibody therapy targeting CD47 and CD271 effectively suppresses melanoma metastasis in patient-derived xenografts. Cell Rep.

[99] Xu J-F, Pan X-H, Zhang S-J (2015). CD47 blockade inhibits tumor progression human osteosarcoma in xenograft models. Oncotarget.

[100] Basset C, Guillermet-Guibert J (2017). Attenuating PI3K isoforms in pancreatic cancer: focus on immune PI3Kγ. Clin Res Hepatol Gastroenterol.

[101] Kaneda MM, Messer KS, Ralainirina N (2016). PI3Kγ is a molecular switch that controls immune suppression. Nature.

[102] De Henau O, Rausch M, Winkler D (2016). Overcoming resistance to checkpoint blockade therapy by targeting PI3Kγ in myeloid cells. Nature.

[103] O’Hayre M, Degese MS, Gutkind JS (2014). Novel insights into G protein and G protein-coupled receptor signaling in cancer. Curr Opin Cell Biol.

[104] Xie Y, Abel PW, Kirui JK (2013). Identification of upregulated phosphoinositide 3-kinase γ as a target to suppress breast cancer cell migration and invasion. Biochem Pharmacol.

[105] Brazzatti JA, Klingler-Hoffmann M, Haylock-Jacobs S (2012). Differential roles for the p101 and p84 regulatory subunits of PI3Kγ in tumor growth and metastasis. Oncogene.

[106] Monterrubio M, Mellado M, Carrera AC, Rodríguez-Frade JM (2009). PI3Kγ activation by CXCL12 regulates tumor cell adhesion and invasion. Biochem Biophys Res Commun.

[107] Han L, Wang W, Lu J (2014). AAV–sBTLA facilitates HSP70 vaccine-triggered prophylactic antitumor immunity against a murine melanoma pulmonary metastasis model in vivo. Cancer Lett.

[108] Sekar D, Govene L, del Río M-L (2018). Downregulation of BTLA on NKT cells promotes tumor immune control in a mouse model of mammary carcinoma. Int J Mol Sci.

[109] Deng W-W, Mao L, Yu G-T (2016). LAG-3 confers poor prognosis and its blockade reshapes antitumor response in head and neck squamous cell carcinoma. Oncoimmunology.

[110] Hemon P, Jean-Louis F, Ramgolam K (2011). MHC class II engagement by its ligand LAG-3 (CD223) contributes to melanoma resistance to apoptosis. J Immunol.

[111] Qin L, Thompson LF, Kuzel TM, Zhang B (2014). Requirement of NK cells for selective A 2A receptor blockade to suppress CD73 + tumor metastasis. Immunotherapy.

[112] Shi L, Wu Z, Miao J (2019). Adenosine interaction with adenosine receptor A2a promotes gastric cancer metastasis by enhancing PI3K–AKT–mTOR signaling. Mol Biol Cell.

[113] Young A, Ngiow SF, Gao Y (2018). A2AR adenosine signaling suppresses natural killer cell maturation in the tumor microenvironment. Cancer Res.

[114] Young A, Ngiow SF, Madore J (2017). Targeting adenosine in braf-mutant melanoma reduces tumor growth and metastasis. Cancer Res.

[115] Kurtulus S, Sakuishi K, Ngiow S-F (2015). TIGIT predominantly regulates the immune response via regulatory T cells. J Clin Invest.

[116] Xu F, Sunderland A, Zhou Y (2017). Blockade of CD112R and TIGIT signaling sensitizes human natural killer cell functions. Cancer Immunol Immunother.

[117] Liu J-F, Ma S-R, Mao L (2017). T-cell immunoglobulin mucin 3 blockade drives an antitumor immune response in head and neck cancer. Mol Oncol.

[118] Wu J, Lin G, Zhu Y (2017). Low TIM3 expression indicates poor prognosis of metastatic prostate cancer and acts as an independent predictor of castration resistant status. Sci Rep.

[119] Gao X, Zhu Y, Li G (2012). TIM-3 expression characterizes regulatory T cells in tumor tissues and is associated with lung cancer progression. PLoS ONE.

[120] Mohammadzadeh R, Baradaran B, Valizadeh H (2014). Reduced ABCB1 expression and activity in the presence of acrylic copolymers. Adv Pharm Bull.

[121] Sun QY, Qu CH, Liu JQ (2017). Down-regulated expression of Tim-3 promotes invasion and metastasis of colorectal cancer cells. Neoplasma.

[122] Wang Y, Sun J, Gao W (2017). Preoperative Tim-3 expression on peripheral NK cells is correlated with pathologic TNM staging in colorectal cancer. Mol Med Rep.

[123] Yu M, Lu B, Liu Y (2017). Tim-3 is upregulated in human colorectal carcinoma and associated with tumor progression. Mol Med Rep.

[124] Yousefi B, Darabi M, Baradaran B (2012). Inhibition of MEK/ERK1/2 signaling affects the fatty acid composition of HepG2 human hepatic cell line. BioImpacts.

[125] Stagg J, Divisekera U, Duret H (2011). CD73-deficient mice have increased antitumor immunity and are resistant to experimental metastasis. Cancer Res.

[126] Burghoff S, Gong X, Viethen C (2014). Growth and metastasis of B16–F10 melanoma cells is not critically dependent on host CD73 expression in mice. BMC Cancer.

[127] Zhang B, Song B, Wang X (2015). The expression and clinical significance of CD73 molecule in human rectal adenocarcinoma. Tumor Biol.

[128] Inoue Y, Yoshimura K, Kurabe N (2017). Prognostic impact of CD73 and A2A adenosine receptor expression in non-small-cell lung cancer. Oncotarget.

[129] Wu X-R, He X-S, Chen Y-F (2012). High expression of CD73 as a poor prognostic biomarker in human colorectal cancer. J Surg Oncol.

[130] Mandapathil M, Boduc M, Netzer C (2018). CD73 expression in lymph node metastases in patients with head and neck cancer. Acta Otolaryngol.

[131] Lee H, Lin ECK, Liu L, Smith JW (2003). Gene expression profiling of tumor xenografts: In vivo analysis of organ-specific metastasis. Int J Cancer.

[132] Yang Q, Du J, Zu L (2013). Overexpression of CD73 in prostate cancer is associated with lymph node metastasis. Pathol Oncol Res.

[133] Monteiro I, Vigano S, Faouzi M (2018). CD73 expression and clinical significance in human metastatic melanoma. Oncotarget.

[134] Lu X-X (2013). Expression and clinical significance of CD73 and hypoxia-inducible factor-1α in gastric carcinoma. World J Gastroenterol.

[135] Zhi X, Wang Y, Zhou X (2010). RNAi-mediated CD73 suppression induces apoptosis and cell-cycle arrest in human breast cancer cells. Cancer Sci.

[136] Bowser JL, Blackburn MR, Shipley GL (2015). Loss of CD73-mediated actin polymerization promotes endometrial tumor progression. J Clin Invest.

[137] Huang R-Y, Francois A, McGray AR (2017). Compensatory upregulation of PD-1, LAG-3, and CTLA-4 limits the efficacy of single-agent checkpoint blockade in metastatic ovarian cancer. Oncoimmunology.

[138] Heidarieh P, Hashemi Shahraki A, Yaghoubfar R (2016). Microbiological analysis of hemodialysis water in a developing country. ASAIO J.

[139] van Elsas A, Hurwitz AA, Allison JP (1999). Combination immunotherapy of B16 melanoma using anti-cytotoxic T lymphocyte-associated antigen 4 (Ctla-4) and granulocyte/macrophage colony-stimulating factor (Gm-Csf)-producing vaccines induces rejection of subcutaneous and metastatic tumors accompanied. J Exp Med.

[140] Kwon ED, Foster BA, Hurwitz AA (1999). Elimination of residual metastatic prostate cancer after surgery and adjunctive cytotoxic T lymphocyte-associated antigen 4 (CTLA-4) blockade immunotherapy. Proc Natl Acad Sci.

[141] Hodi FS, Oble DA, Drappatz J (2008). CTLA-4 blockade with ipilimumab induces significant clinical benefit in a female with melanoma metastases to the CNS. Nat Clin Pract Oncol.

[142] Phan GQ, Weber JS, Sondak VK (2008). CTLA-4 blockade with monoclonal antibodies in patients with metastatic cancer: surgical issues. Ann Surg Oncol.

[143] Yang JC, Hughes M, Kammula U (2007). Ipilimumab (anti-CTLA4 antibody) causes regression of metastatic renal cell cancer associated with enteritis and hypophysitis. J Immunother.

[144] Graff JN, Puri S, Bifulco CB (2014). Sustained complete response to CTLA-4 blockade in a patient with metastatic, castration-resistant prostate cancer. Cancer Immunol Res.

[145] Kakavand H, Vilain RE, Wilmott JS (2015). Tumor PD-L1 expression, immune cell correlates and PD-1+ lymphocytes in sentinel lymph node melanoma metastases. Mod Pathol.

[146] Anantharaman A, Friedlander T, Lu D (2016). Programmed death-ligand 1 (PD-L1) characterization of circulating tumor cells (CTCs) in muscle invasive and metastatic bladder cancer patients. BMC Cancer.

[147] Obeid JM, Erdag G, Smolkin ME (2016). PD-L1, PD-L2 and PD-1 expression in metastatic melanoma: correlation with tumor-infiltrating immune cells and clinical outcome. Oncoimmunology.

[148] Callea M, Albiges L, Gupta M (2015). Differential expression of PD-L1 between primary and metastatic sites in clear-cell renal cell carcinoma. Cancer Immunol Res.

[149] Duchnowska R, Pęksa R, Radecka B (2016). Immune response in breast cancer brain metastases and their microenvironment: the role of the PD-1/PD-L axis. Breast Cancer Res.

[150] Kim ST, Ha SY, Lee S (2016). The impact of PD-L1 expression in patients with metastatic GEP-NETs. J Cancer.

[151] Wu C, Zhu Y, Jiang J (2006). Immunohistochemical localization of programmed death-1 ligand-1 (PD-L1) in gastric carcinoma and its clinical significance. Acta Histochem.

[152] Jilaveanu LB, Shuch B, Zito CR (2014). PD-L1 expression in clear cell renal cell carcinoma: an analysis of nephrectomy and sites of metastases. J Cancer.

[153] Lussier DM, O’Neill L, Nieves LM (2015). Enhanced T cell immunity to osteosarcoma through antibody blockade of PD-1/PD-L1 interactions. J Immunother.

[154] Lin Y-M, Sung W-W, Hsieh M-J (2015). High PD-L1 expression correlates with metastasis and poor prognosis in oral squamous cell carcinoma. PLoS ONE.

[155] Ukpo OC, Thorstad WL, Lewis JS (2013). B7–H1 Expression model for immune evasion in human papillomavirus-related oropharyngeal squamous cell carcinoma. Head Neck Pathol.

[156] Thompson RH, Kwon ED (2006). Significance of B7–H1 Overexpression in Kidney Cancer. Clin Genitourin Cancer.

[157] Wu C-P, Jiang J-T, Tan M (2006). Relationship between co-stimulatory molecule B7–H3 expression and gastric carcinoma histology and prognosis. World J Gastroenterol.

[158] Chen Y, Zhao H, Zhu D (2014). The coexpression and clinical significance of costimulatory molecules B7–H1, B7–H3, and B7–H4 in human pancreatic cancer. Onco Targets Ther.

[159] Song X, Tang T, Li C (2018). CBX8 and CD96 are important prognostic biomarkers of colorectal cancer. Med Sci Monit.

[160] Zhang G, Xu Y, Lu X (2009). Diagnosis value of serum B7–H3 expression in non-small cell lung cancer. Lung Cancer.

[161] Tan M, Zhu L, Zhuang H (2015). Lewis Y antigen modified CD47 is an independent risk factor for poor prognosis and promotes early ovarian cancer metastasis. Am J Cancer Res.

[162] Baccelli I, Stenzinger A, Vogel V (2014). Co-expression of MET and CD47 is a novel prognosticator for survival of luminal-type breast cancer patients. Oncotarget.

[163] Fujiwara-Tani R, Sasaki T, Ohmori H (2019). Concurrent expression of CD47 and CD44 in colorectal cancer promotes malignancy. Pathobiology.

[164] Karami H, Baradaran B, Esfahani A (2013). siRNA-mediated silencing of survivin inhibits proliferation and enhances etoposide chemosensitivity in acute myeloid leukemia cells. Asian Pac J Cancer Prev.

[165] Hotson A, Powderly J, Emens L, et al (2020) Clinical activity of adenosine 2A receptor (A2AR) inhibitor CPI-444 is associated with tumor expression of adenosine pathway genes and tumor immune modulation. In: Society for Immunotherapy of Cancer Annual Meeting

[166] Mediavilla-Varela M, Castro J, Chiappori A (2017). A novel antagonist of the immune checkpoint protein adenosine A2a receptor restores tumor-infiltrating lymphocyte activity in the context of the tumor microenvironment. Neoplasia.

[167] Lan X, Li S, Gao H (2017). Increased BTLA and HVEM in gastric cancer are associated with progression and poor prognosis. Oncol Targets Ther.

[168] Geng H, Chen Z, Anderson S (2015). Expression of B and T lymphocyte attenuator (BTLA) correlates with CNS metastasis and adverse prognosis in activated B-Cell lymphoma and acute lymphoblastic leukemia. Blood.

[169] Hald SM, Rakaee M, Martinez I (2018). LAG-3 in non–small-cell lung cancer: expression in primary tumors and metastatic lymph nodes is associated with improved survival. Clin Lung Cancer.

[170] Wu L, Deng W-W, Huang C-F (2017). Expression of VISTA correlated with immunosuppression and synergized with CD8 to predict survival in human oral squamous cell carcinoma. Cancer Immunol Immunother.

[171] Kakavand H, Jackett LA, Menzies AM (2017). Negative immune checkpoint regulation by VISTA: a mechanism of acquired resistance to anti-PD-1 therapy in metastatic melanoma patients. Mod Pathol.

[172] Baumgartner J, Wilson C, Palmer B (2007). Melanoma induces immunosuppression by up-regulating FOXP3+ regulatory T cells. J Surg Res.

[173] Villano JL, Durbin EB, Normandeau C (2015). Incidence of brain metastasis at initial presentation of lung cancer. Neuro Oncol.

[174] Turkoz FP, Solak M, Kilickap S (2014). Bone metastasis from gastric cancer: the incidence, clinicopathological features, and influence on survival. J Gastric Cancer.

